# 2-(1-Ethyl-5-meth­oxy-1*H*-indol-3-yl)-*N*-(4-meth­oxy­phen­yl)-2-oxoacetamide

**DOI:** 10.1107/S1600536810054668

**Published:** 2011-01-12

**Authors:** Li-Ting Chen, Yan-Ling Lu, Hong Chen, Jing Zhou

**Affiliations:** a School of Pharmacy, Tianjin Medical University, Tianjin 300070, People’s Republic of China; b Room of Pharmacognosy, Medical College of Chinese People’s Armed Police Forces, Tianjin 300162, People’s Republic of China; c Tianjin Key Laboratory for Biomarkers of Occupational and Environmental Hazards, Tianjin 300162, People’s Republic of China

## Abstract

The title compound, C_20_H_20_N_2_O_4_, crystallizes with four independent mol­ecules in the asymmetric unit. In the mol­ecules, the dihedral angles between the benzene rings and indole mean planes are 24.5 (1), 22.5 (1), 8.8 (1) and 13.9 (1)°. In the crystal, inter­molecular N—H⋯O hydrogen bonds are present between the imino groups and the adjacent carbonyl groups. π–π stacking is also observed with a centroid–centroid distance between nearly parallel pyrrole rings of 3.745 (3) Å.

## Related literature

For the biological activity of the title compound and related compounds, see: Souli *et al.* (2008 ▶); Liu *et al.* (2007 ▶); Chai *et al.* (2006 ▶); Radwan *et al.* (2007 ▶); Karthikeyan *et al.* (2009 ▶). For the preparation, see: Bacher *et al.* (2001 ▶).
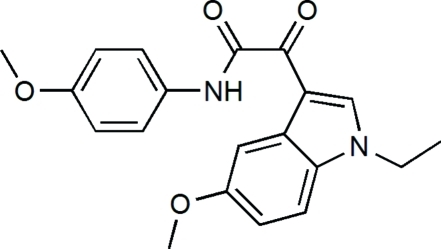


## Experimental

### 

#### Crystal data

C_20_H_20_N_2_O_4_
*M* *_r_* = 352.38
Monoclinic, 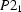

*a* = 8.3622 (17) Å
*b* = 35.073 (7) Å
*c* = 12.280 (3) Å
β = 105.40 (3)°
*V* = 3472.1 (14) Å^3^
*Z* = 8
Mo *K*α radiation
μ = 0.10 mm^−1^
*T* = 153 K
0.20 × 0.14 × 0.08 mm

#### Data collection

Rigaku Saturn CCD area-detector diffractometer
33298 measured reflections
7700 independent reflections
6293 reflections with *I* > 2σ(*I*)
*R*_int_ = 0.075

#### Refinement

*R*[*F* ^2^ > 2σ(*F* ^2^)] = 0.062
*wR*(*F* ^2^) = 0.144
*S* = 1.09
7700 reflections
966 parameters
3 restraints
H atoms treated by a mixture of independent and constrained refinement
Δρ_max_ = 0.34 e Å^−3^
Δρ_min_ = −0.29 e Å^−3^

### 

Data collection: *CrystalClear* (Rigaku/MSC, 2005 ▶); cell refinement: *CrystalClear*; data reduction: *CrystalStructure* (Rigaku/MSC, 2005 ▶); program(s) used to solve structure: *SHELXTL* (Sheldrick, 2008 ▶); program(s) used to refine structure: *SHELXTL*; molecular graphics: *SHELXTL*; software used to prepare material for publication: *SHELXTL*.

## Supplementary Material

Crystal structure: contains datablocks I, global. DOI: 10.1107/S1600536810054668/xu5111sup1.cif

Structure factors: contains datablocks I. DOI: 10.1107/S1600536810054668/xu5111Isup2.hkl

Additional supplementary materials:  crystallographic information; 3D view; checkCIF report

## Figures and Tables

**Table 1:** Hydrogen-bond geometry (Å, °)

*D*—H⋯*A*	*D*—H	H⋯*A*	*D*⋯*A*	*D*—H⋯*A*
N2—H2⋯O6^i^	0.88 (3)	2.17 (3)	2.967 (5)	150 (4)
N4—H4*C*⋯O2^ii^	0.85 (5)	2.44 (5)	3.245 (5)	159 (5)
N6—H6⋯O14^iii^	0.96 (5)	2.30 (5)	3.196 (5)	154 (4)
N8—H8⋯O10^iv^	0.92 (3)	2.08 (4)	2.936 (5)	155 (7)

Symmetry codes: (i) 
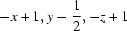
; (ii) 
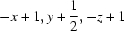
; (iii) 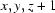; (iv) 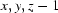.

**Fig. 1.**
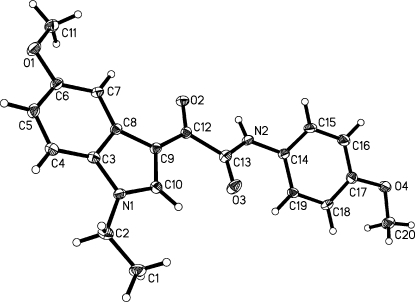
The molecular structure and atom-labeling scheme of (I).

**Fig. 2.**
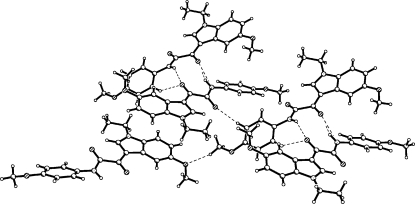
The N—H···O hydrogen bonds stabling the packing structure of (I). Hydrogen bonds are shown as dashed lines.

## supplementary crystallographic information

### Comment

The indole substructure is a basic core unit for numerous physiologically active natural and synthetic molecule, hence indole and its derivatives always act as lead compounds in many pharmaceutical with variety of biological activity such as anti-cancer (Souli *et al.*, 2008), anti-thrombosis (Liu *et al.*, 2007), anti-tubercular (Karthikeyan *et al.*, 2009), anti-virus(Chai *et al.*, 2006), and anti-inflammatory(Radwan *et al*., 2007).

In this work, the title compound (I), C_20_H_20_N_2_O_4_, (Fig.1), has been synthesized. In an asymmetric unit of (I) four molecules can be observed. (I) crystallizes in the Monoclinic,P2(1) space group, a = 8.3622 (17) Å, b = 35.074 (7) Å, c = 12.280 (3) Å, β = 105.40 (3)°. The dihedral angle between the anisole and indole planes is 24.5 (1)°, 22.5 (1)°, 8.8 (1)° and 13.9 (1)°. N atoms in the molecule act as hydrogen-bond donors to O atoms in the adjacent molecules forming intermolecular N2—H2···O6 (symmetry code: -*x* + 1, *y* - 1/2, -*z* + 1), N4—H4C···O2 (symmetry code: -*x* + 1, *y* + 1/2, -*z* + 1), N6—H6···O14 (symmetry code: *x*, *y*, *z* + 1) and N8—H8···O10 (symmetry code: *x*, *y*, *z* - 1) hydrogen bonds, and these N—H···O hydrogen bonds stabilize the crystal structure.π-π interactions between the indole rings are also present, and the centroid-centroid distance between the adjacent pyrrole rings is 3.745 (1) Å. The parallel slipped π-π interactions between the indole rings further consolidate (I) into the three-dimensional supramolecular architecture.

Perspective drawing with the atomic numbering scheme is showed in Figure1. The N—H···O hydrogen bonds are illustrated in Figure 2.

### Experimental

The target compound was synthesized following the method described by Bacher *et al.* (2001). Reaction of treated 5-methoxyl-indole (by NaH) with bromoethane in dimethylformamide yielded 1-ethyl-5-methoxy-1*H*-indole in 85% yield. Treatment of 1-ethyl-5-methoxy-1*H*-indole with oxalylchloride in dry ether as solvent, the target compound was synthesized by the reaction of 2-(1-ethyl-5-methoxy-1*H*-indol-3-yl)-2-oxoacetyl chloride with 4-methoxybenzenamine in dry DCM in the presence of triethylamine. Yellow prism crystals were obtained by slow evaporation from a methanol solution of product at room temperature.

### Refinement

Imino H atoms were located in a difference Fourier map and refined isotropically. Other H atoms were positioned geometrically and refined by a riding model, with C—H = 0.95-0.99 Å and U_iso_(H) = 1.5U_eq_(C) for methyl and 1.2U_eq_(C) for the others. As no significant anomalous scatterings, Friedel pairs were merged.

### Crystal data

**Table:**

C_20_H_20_N_2_O_4_	*F*(000) = 1488
*M**_r_* = 352.38	*D*_x_ = 1.348 Mg m^−^^3^
Monoclinic, *P*2_1_	Mo *K*α radiation, λ = 0.71073 Å
Hall symbol: P 2yb	Cell parameters from 7641 reflections
*a* = 8.3622 (17) Å	θ = 1.7–27.9°
*b* = 35.073 (7) Å	µ = 0.10 mm^−^^1^
*c* = 12.280 (3) Å	*T* = 153 K
β = 105.40 (3)°	Block, yellow
*V* = 3472.1 (14) Å^3^	0.20 × 0.14 × 0.08 mm
*Z* = 8	

### Data collection

**Table:**

Rigaku Saturn CCD area-detector diffractometer	6293 reflections with *I* > 2σ(*I*)
Radiation source: rotating anode	*R*_int_ = 0.075
multilayer	θ_max_ = 27.0°, θ_min_ = 1.7°
Detector resolution: 7.31 pixels mm^-1^	*h* = −10→10
ω and φ scans	*k* = −44→44
33298 measured reflections	*l* = −15→15
7700 independent reflections	

### Refinement

**Table:**

Refinement on *F*^2^	Secondary atom site location: difference Fourier map
Least-squares matrix: full	Hydrogen site location: inferred from neighbouring sites
*R*[*F*^2^ > 2σ(*F*^2^)] = 0.062	H atoms treated by a mixture of independent and constrained refinement
*wR*(*F*^2^) = 0.144	*w* = 1/[σ^2^(*F*_o_^2^) + (0.0684*P*)^2^ + 0.2586*P*] where *P* = (*F*_o_^2^ + 2*F*_c_^2^)/3
*S* = 1.09	(Δ/σ)_max_ < 0.001
7700 reflections	Δρ_max_ = 0.34 e Å^−^^3^
966 parameters	Δρ_min_ = −0.29 e Å^−^^3^
3 restraints	Extinction correction: *SHELXTL* (Sheldrick, 2008), Fc^*^=kFc[1+0.001xFc^2^λ^3^/sin(2θ)]^-1/4^
Primary atom site location: structure-invariant direct methods	Extinction coefficient: 0.0172 (11)

### Special details

**Table:**

Geometry. All e.s.d.'s (except the e.s.d. in the dihedral angle between two l.s. planes) are estimated using the full covariance matrix. The cell e.s.d.'s are taken into account individually in the estimation of e.s.d.'s in distances, angles and torsion angles; correlations between e.s.d.'s in cell parameters are only used when they are defined by crystal symmetry. An approximate (isotropic) treatment of cell e.s.d.'s is used for estimating e.s.d.'s involving l.s. planes.
Refinement. Refinement of *F*^2^ against ALL reflections. The weighted *R*-factor *wR* and goodness of fit *S* are based on *F*^2^, conventional *R*-factors *R* are based on *F*, with *F* set to zero for negative *F*^2^. The threshold expression of *F*^2^ > σ(*F*^2^) is used only for calculating *R*-factors(gt) *etc*. and is not relevant to the choice of reflections for refinement. *R*-factors based on *F*^2^ are statistically about twice as large as those based on *F*, and *R*- factors based on ALL data will be even larger.

### Fractional atomic coordinates and isotropic or equivalent isotropic displacement parameters (Å^2^)

**Table:**

	*x*	*y*	*z*	*U*_iso_*/*U*_eq_	
O1	0.2676 (4)	0.35006 (9)	0.5466 (3)	0.0370 (8)	
O2	0.5650 (4)	0.23984 (8)	0.3740 (2)	0.0275 (7)	
O3	0.5068 (4)	0.22202 (10)	0.0856 (3)	0.0400 (8)	
O4	0.9534 (5)	0.07562 (9)	0.0456 (3)	0.0453 (9)	
O5	−0.3578 (4)	0.65181 (10)	0.7125 (3)	0.0406 (8)	
O6	0.1229 (4)	0.68191 (9)	0.5252 (3)	0.0339 (7)	
O7	0.1701 (4)	0.64865 (9)	0.2659 (3)	0.0342 (8)	
O8	0.8669 (4)	0.72400 (9)	0.2329 (3)	0.0332 (8)	
O9	−0.2589 (4)	0.47778 (10)	1.1870 (3)	0.0446 (9)	
O10	0.2161 (4)	0.44383 (9)	0.9981 (3)	0.0320 (7)	
O11	0.2559 (4)	0.47258 (9)	0.7329 (3)	0.0340 (7)	
O12	0.9539 (4)	0.39720 (9)	0.7060 (3)	0.0335 (7)	
O13	0.8268 (4)	0.27449 (9)	−0.0748 (3)	0.0369 (8)	
O14	0.5241 (4)	0.38500 (8)	0.0956 (3)	0.0281 (7)	
O15	0.5974 (4)	0.40510 (9)	0.3842 (3)	0.0379 (8)	
O16	0.1803 (4)	0.55424 (9)	0.4334 (3)	0.0361 (8)	
N1	0.2514 (4)	0.31999 (9)	0.1045 (3)	0.0247 (8)	
N2	0.6751 (4)	0.19332 (10)	0.2390 (3)	0.0249 (8)	
H2	0.701 (6)	0.1933 (14)	0.313 (2)	0.034 (13)*	
N3	−0.2643 (4)	0.60163 (10)	0.3095 (3)	0.0301 (8)	
N4	0.3120 (4)	0.69362 (11)	0.3896 (3)	0.0270 (8)	
H4C	0.317 (6)	0.7072 (16)	0.447 (4)	0.043 (15)*	
N5	−0.2051 (5)	0.51051 (10)	0.7612 (4)	0.0351 (9)	
N6	0.3964 (4)	0.42794 (10)	0.8588 (3)	0.0245 (8)	
H6	0.404 (5)	0.4190 (12)	0.934 (4)	0.025 (12)*	
N7	0.8389 (4)	0.30499 (10)	0.3659 (3)	0.0270 (8)	
N8	0.4174 (5)	0.43227 (10)	0.2310 (3)	0.0272 (8)	
H8	0.373 (9)	0.429 (2)	0.155 (3)	0.11 (3)*	
C1	0.2142 (7)	0.32665 (16)	−0.1015 (4)	0.0455 (13)	
H1A	0.3345	0.3287	−0.0897	0.068*	
H1B	0.1577	0.3421	−0.1670	0.068*	
H1C	0.1803	0.2999	−0.1150	0.068*	
C2	0.1684 (5)	0.34089 (13)	0.0018 (4)	0.0294 (10)	
H2A	0.1977	0.3682	0.0127	0.035*	
H2B	0.0468	0.3387	−0.0106	0.035*	
C3	0.2391 (5)	0.33069 (12)	0.2125 (4)	0.0245 (9)	
C4	0.1517 (5)	0.36062 (12)	0.2425 (4)	0.0282 (9)	
H4	0.0858	0.3772	0.1873	0.034*	
C5	0.1647 (5)	0.36529 (12)	0.3559 (4)	0.0298 (10)	
H5	0.1055	0.3853	0.3801	0.036*	
C6	0.2640 (6)	0.34086 (12)	0.4358 (4)	0.0294 (10)	
C7	0.3483 (5)	0.31046 (12)	0.4064 (4)	0.0262 (9)	
H7	0.4125	0.2937	0.4619	0.031*	
C8	0.3356 (5)	0.30525 (11)	0.2915 (4)	0.0243 (9)	
C9	0.4047 (5)	0.27787 (11)	0.2280 (4)	0.0241 (9)	
C10	0.3485 (5)	0.28893 (11)	0.1149 (4)	0.0254 (9)	
H10	0.3752	0.2762	0.0536	0.030*	
C11	0.3848 (6)	0.33059 (14)	0.6342 (4)	0.0401 (12)	
H11A	0.3613	0.3032	0.6286	0.060*	
H11B	0.3769	0.3400	0.7077	0.060*	
H11C	0.4969	0.3352	0.6265	0.060*	
C12	0.5130 (5)	0.24686 (11)	0.2714 (4)	0.0246 (9)	
C13	0.5644 (5)	0.21948 (12)	0.1875 (4)	0.0266 (9)	
C14	0.7403 (5)	0.16398 (12)	0.1846 (4)	0.0252 (9)	
C15	0.7809 (6)	0.12927 (13)	0.2390 (4)	0.0363 (11)	
H15	0.7608	0.1253	0.3107	0.044*	
C16	0.8500 (6)	0.10045 (13)	0.1902 (4)	0.0407 (12)	
H16	0.8753	0.0767	0.2279	0.049*	
C17	0.8826 (6)	0.10587 (12)	0.0873 (4)	0.0330 (10)	
C18	0.8397 (5)	0.14005 (12)	0.0294 (4)	0.0279 (9)	
H18	0.8591	0.1437	−0.0427	0.033*	
C19	0.7680 (5)	0.16885 (11)	0.0785 (4)	0.0249 (9)	
H19	0.7374	0.1922	0.0391	0.030*	
C20	1.0287 (7)	0.08369 (14)	−0.0422 (4)	0.0406 (11)	
H20A	0.9444	0.0929	−0.1087	0.061*	
H20B	1.0800	0.0605	−0.0618	0.061*	
H20C	1.1138	0.1033	−0.0170	0.061*	
C21	−0.5032 (6)	0.59621 (15)	0.1414 (4)	0.0459 (13)	
H21A	−0.5730	0.6086	0.1837	0.069*	
H21B	−0.5698	0.5778	0.0882	0.069*	
H21C	−0.4585	0.6155	0.0997	0.069*	
C22	−0.3609 (5)	0.57555 (12)	0.2232 (4)	0.0339 (10)	
H22A	−0.4059	0.5547	0.2606	0.041*	
H22B	−0.2874	0.5641	0.1808	0.041*	
C23	−0.3092 (5)	0.61298 (12)	0.4071 (4)	0.0267 (10)	
C24	−0.4523 (5)	0.60501 (12)	0.4397 (4)	0.0326 (10)	
H24	−0.5398	0.5903	0.3933	0.039*	
C25	−0.4622 (5)	0.61941 (13)	0.5427 (4)	0.0338 (11)	
H25	−0.5588	0.6147	0.5674	0.041*	
C26	−0.3320 (6)	0.64087 (13)	0.6115 (4)	0.0323 (10)	
C27	−0.1929 (6)	0.64974 (13)	0.5770 (4)	0.0309 (10)	
H27	−0.1066	0.6648	0.6228	0.037*	
C28	−0.1824 (5)	0.63569 (12)	0.4714 (4)	0.0250 (9)	
C29	−0.0601 (5)	0.63950 (12)	0.4078 (4)	0.0251 (9)	
C30	−0.1187 (5)	0.61774 (12)	0.3104 (4)	0.0278 (10)	
H30	−0.0632	0.6148	0.2527	0.033*	
C31	−0.2295 (6)	0.67355 (17)	0.7853 (4)	0.0458 (13)	
H31A	−0.1265	0.6587	0.8044	0.069*	
H31B	−0.2611	0.6797	0.8547	0.069*	
H31C	−0.2126	0.6972	0.7473	0.069*	
C32	0.0829 (5)	0.66360 (12)	0.4354 (4)	0.0257 (9)	
C33	0.1925 (5)	0.66782 (12)	0.3528 (4)	0.0250 (9)	
C34	0.4458 (5)	0.70202 (12)	0.3424 (4)	0.0243 (9)	
C35	0.5888 (5)	0.71758 (12)	0.4127 (4)	0.0293 (10)	
H35	0.5928	0.7233	0.4890	0.035*	
C36	0.7241 (5)	0.72468 (13)	0.3730 (4)	0.0301 (10)	
H36	0.8213	0.7352	0.4222	0.036*	
C37	0.7214 (5)	0.71674 (12)	0.2622 (4)	0.0276 (9)	
C38	0.5790 (5)	0.70241 (13)	0.1899 (4)	0.0285 (10)	
H38	0.5748	0.6975	0.1131	0.034*	
C39	0.4409 (5)	0.69508 (13)	0.2303 (4)	0.0301 (10)	
H39	0.3425	0.6852	0.1804	0.036*	
C40	0.8756 (6)	0.71101 (14)	0.1233 (4)	0.0344 (11)	
H40A	0.8544	0.6835	0.1169	0.052*	
H40B	0.9862	0.7163	0.1139	0.052*	
H40C	0.7920	0.7244	0.0646	0.052*	
C41	−0.2906 (6)	0.51840 (16)	0.5528 (5)	0.0509 (14)	
H41A	−0.1891	0.5322	0.5516	0.076*	
H41B	−0.3827	0.5275	0.4910	0.076*	
H41C	−0.2742	0.4911	0.5434	0.076*	
C42	−0.3300 (7)	0.52523 (17)	0.6639 (5)	0.0546 (15)	
H42A	−0.3421	0.5530	0.6738	0.065*	
H42B	−0.4379	0.5132	0.6614	0.065*	
C43	−0.2343 (6)	0.50552 (13)	0.8674 (4)	0.0344 (11)	
C44	−0.3746 (6)	0.51447 (14)	0.9024 (5)	0.0415 (13)	
H44	−0.4667	0.5271	0.8535	0.050*	
C45	−0.3754 (6)	0.50445 (14)	1.0102 (5)	0.0400 (12)	
H45	−0.4694	0.5103	1.0368	0.048*	
C46	−0.2401 (6)	0.48580 (13)	1.0816 (4)	0.0346 (11)	
C47	−0.0983 (5)	0.47683 (12)	1.0474 (4)	0.0298 (10)	
H47	−0.0063	0.4644	1.0969	0.036*	
C48	−0.0974 (5)	0.48684 (12)	0.9376 (4)	0.0293 (10)	
C49	0.0201 (5)	0.48122 (12)	0.8707 (4)	0.0274 (9)	
C50	−0.0529 (5)	0.49650 (12)	0.7642 (4)	0.0321 (10)	
H50	−0.0033	0.4970	0.7030	0.039*	
C51	−0.1286 (7)	0.45760 (18)	1.2635 (5)	0.0499 (14)	
H51A	−0.1160	0.4324	1.2323	0.075*	
H51B	−0.1553	0.4546	1.3361	0.075*	
H51C	−0.0247	0.4719	1.2748	0.075*	
C52	0.1695 (5)	0.45955 (12)	0.9046 (4)	0.0250 (9)	
C53	0.2770 (5)	0.45444 (12)	0.8207 (4)	0.0263 (9)	
C54	0.5322 (5)	0.41958 (12)	0.8122 (4)	0.0252 (9)	
C55	0.6768 (5)	0.40588 (12)	0.8860 (4)	0.0250 (9)	
H55	0.6815	0.4016	0.9632	0.030*	
C56	0.8143 (5)	0.39852 (12)	0.8462 (4)	0.0274 (9)	
H56	0.9136	0.3894	0.8967	0.033*	
C57	0.8079 (5)	0.40429 (11)	0.7333 (4)	0.0242 (9)	
C58	0.6621 (5)	0.41653 (13)	0.6597 (4)	0.0313 (10)	
H58	0.6561	0.4198	0.5819	0.038*	
C59	0.5236 (5)	0.42412 (13)	0.6989 (4)	0.0288 (10)	
H59	0.4232	0.4324	0.6479	0.035*	
C60	0.9638 (6)	0.40860 (14)	0.5966 (4)	0.0335 (10)	
H60A	0.8780	0.3954	0.5389	0.050*	
H60B	1.0734	0.4020	0.5873	0.050*	
H60C	0.9468	0.4362	0.5881	0.050*	
C61	0.8703 (7)	0.29648 (15)	0.5721 (4)	0.0457 (13)	
H61A	0.8993	0.3234	0.5881	0.068*	
H61B	0.9285	0.2809	0.6367	0.068*	
H61C	0.7503	0.2932	0.5591	0.068*	
C62	0.9207 (5)	0.28422 (13)	0.4685 (4)	0.0314 (10)	
H62A	1.0421	0.2875	0.4829	0.038*	
H62B	0.8961	0.2567	0.4557	0.038*	
C63	0.8519 (5)	0.29486 (11)	0.2582 (4)	0.0259 (9)	
C64	0.9429 (5)	0.26509 (12)	0.2291 (4)	0.0296 (10)	
H64	1.0090	0.2487	0.2849	0.036*	
C65	0.9332 (5)	0.26033 (12)	0.1167 (4)	0.0307 (10)	
H65	0.9965	0.2408	0.0941	0.037*	
C66	0.8305 (5)	0.28394 (12)	0.0342 (4)	0.0280 (9)	
C67	0.7432 (5)	0.31428 (11)	0.0642 (4)	0.0259 (9)	
H67	0.6776	0.3307	0.0084	0.031*	
C68	0.7552 (5)	0.31982 (11)	0.1786 (4)	0.0230 (9)	
C69	0.6870 (5)	0.34735 (11)	0.2434 (4)	0.0232 (9)	
C70	0.7417 (5)	0.33617 (12)	0.3569 (4)	0.0259 (9)	
H70	0.7145	0.3487	0.4182	0.031*	
C71	0.7026 (7)	0.29181 (14)	−0.1630 (4)	0.0411 (12)	
H71A	0.5940	0.2886	−0.1480	0.062*	
H71B	0.7015	0.2797	−0.2352	0.062*	
H71C	0.7267	0.3191	−0.1667	0.062*	
C72	0.5792 (5)	0.37856 (11)	0.1973 (4)	0.0227 (9)	
C73	0.5321 (5)	0.40614 (12)	0.2821 (4)	0.0249 (9)	
C74	0.3592 (5)	0.46213 (11)	0.2875 (4)	0.0248 (9)	
C75	0.3134 (5)	0.49667 (12)	0.2300 (4)	0.0279 (9)	
H75	0.3232	0.4996	0.1551	0.033*	
C76	0.2545 (5)	0.52641 (12)	0.2815 (4)	0.0282 (9)	
H76	0.2240	0.5497	0.2419	0.034*	
C77	0.2392 (5)	0.52271 (12)	0.3907 (4)	0.0271 (9)	
C78	0.2814 (5)	0.48850 (12)	0.4485 (4)	0.0270 (9)	
H78	0.2699	0.4857	0.5230	0.032*	
C79	0.3410 (5)	0.45819 (12)	0.3957 (4)	0.0269 (9)	
H79	0.3692	0.4347	0.4347	0.032*	
C80	0.1535 (7)	0.55112 (14)	0.5417 (4)	0.0413 (12)	
H80A	0.0839	0.5288	0.5439	0.062*	
H80B	0.0976	0.5741	0.5580	0.062*	
H80C	0.2602	0.5482	0.5982	0.062*	

### Atomic displacement parameters (Å^2^)

**Table:**

	*U*^11^	*U*^22^	*U*^33^	*U*^12^	*U*^13^	*U*^23^
O1	0.044 (2)	0.0437 (19)	0.0252 (18)	0.0066 (15)	0.0130 (16)	−0.0047 (14)
O2	0.0276 (16)	0.0324 (15)	0.0232 (16)	0.0029 (12)	0.0080 (13)	0.0046 (12)
O3	0.0404 (19)	0.052 (2)	0.0238 (19)	0.0173 (16)	0.0017 (15)	−0.0038 (15)
O4	0.074 (3)	0.0297 (16)	0.048 (2)	0.0150 (16)	0.042 (2)	0.0059 (15)
O5	0.0387 (19)	0.055 (2)	0.035 (2)	0.0012 (16)	0.0217 (16)	0.0006 (16)
O6	0.0289 (16)	0.0479 (19)	0.0269 (18)	−0.0066 (14)	0.0111 (14)	−0.0054 (14)
O7	0.0346 (18)	0.0456 (18)	0.0255 (18)	−0.0077 (14)	0.0133 (15)	−0.0097 (14)
O8	0.0287 (17)	0.0458 (18)	0.0310 (19)	−0.0108 (14)	0.0182 (15)	−0.0044 (14)
O9	0.038 (2)	0.057 (2)	0.047 (2)	−0.0083 (16)	0.0251 (18)	−0.0098 (18)
O10	0.0308 (16)	0.0468 (19)	0.0184 (17)	0.0064 (14)	0.0063 (14)	0.0037 (13)
O11	0.0290 (17)	0.0442 (18)	0.0320 (19)	0.0065 (14)	0.0139 (15)	0.0087 (15)
O12	0.0259 (16)	0.0475 (18)	0.0305 (18)	0.0069 (14)	0.0137 (14)	−0.0002 (14)
O13	0.046 (2)	0.0392 (18)	0.0290 (19)	0.0093 (15)	0.0165 (16)	−0.0047 (14)
O14	0.0319 (17)	0.0341 (16)	0.0194 (16)	0.0047 (13)	0.0086 (14)	0.0008 (12)
O15	0.0440 (19)	0.0484 (19)	0.0179 (17)	0.0190 (15)	0.0023 (15)	−0.0056 (14)
O16	0.0445 (19)	0.0301 (16)	0.038 (2)	0.0086 (14)	0.0191 (16)	−0.0007 (14)
N1	0.0297 (19)	0.0272 (18)	0.0185 (19)	0.0043 (14)	0.0085 (15)	0.0025 (14)
N2	0.0272 (19)	0.0307 (18)	0.0188 (19)	0.0049 (14)	0.0097 (16)	0.0017 (15)
N3	0.0267 (19)	0.0290 (18)	0.034 (2)	−0.0033 (15)	0.0077 (17)	−0.0064 (16)
N4	0.0238 (19)	0.033 (2)	0.025 (2)	−0.0021 (15)	0.0090 (16)	−0.0043 (16)
N5	0.030 (2)	0.035 (2)	0.039 (2)	0.0093 (16)	0.0064 (18)	0.0054 (17)
N6	0.0249 (19)	0.0305 (18)	0.021 (2)	0.0033 (14)	0.0113 (16)	0.0031 (15)
N7	0.0259 (18)	0.0300 (18)	0.027 (2)	0.0014 (15)	0.0096 (16)	0.0046 (15)
N8	0.031 (2)	0.0270 (18)	0.025 (2)	0.0040 (15)	0.0095 (17)	−0.0015 (15)
C1	0.057 (3)	0.051 (3)	0.028 (3)	0.020 (3)	0.009 (2)	0.012 (2)
C2	0.026 (2)	0.035 (2)	0.026 (2)	−0.0014 (18)	0.0045 (19)	0.0070 (18)
C3	0.020 (2)	0.027 (2)	0.026 (2)	−0.0003 (16)	0.0058 (18)	0.0022 (17)
C4	0.024 (2)	0.030 (2)	0.032 (3)	0.0027 (17)	0.011 (2)	0.0060 (18)
C5	0.029 (2)	0.028 (2)	0.037 (3)	0.0030 (17)	0.016 (2)	−0.0022 (19)
C6	0.034 (2)	0.031 (2)	0.027 (2)	0.0002 (18)	0.015 (2)	−0.0046 (18)
C7	0.030 (2)	0.031 (2)	0.019 (2)	−0.0006 (17)	0.0092 (18)	0.0018 (17)
C8	0.023 (2)	0.026 (2)	0.027 (2)	0.0003 (16)	0.0108 (18)	−0.0010 (17)
C9	0.022 (2)	0.026 (2)	0.026 (2)	0.0016 (16)	0.0082 (18)	0.0000 (17)
C10	0.023 (2)	0.026 (2)	0.026 (2)	0.0027 (16)	0.0060 (19)	0.0032 (17)
C11	0.056 (3)	0.042 (3)	0.021 (2)	0.006 (2)	0.009 (2)	0.000 (2)
C12	0.025 (2)	0.026 (2)	0.023 (2)	0.0000 (16)	0.0074 (18)	0.0001 (17)
C13	0.022 (2)	0.031 (2)	0.026 (2)	0.0027 (17)	0.0051 (18)	−0.0017 (18)
C14	0.026 (2)	0.029 (2)	0.022 (2)	0.0019 (17)	0.0093 (18)	0.0005 (17)
C15	0.049 (3)	0.035 (2)	0.033 (3)	0.009 (2)	0.024 (2)	0.007 (2)
C16	0.058 (3)	0.030 (2)	0.042 (3)	0.013 (2)	0.029 (3)	0.010 (2)
C17	0.038 (2)	0.028 (2)	0.038 (3)	0.0051 (18)	0.020 (2)	−0.0013 (19)
C18	0.032 (2)	0.033 (2)	0.022 (2)	0.0014 (18)	0.0138 (19)	−0.0022 (17)
C19	0.030 (2)	0.024 (2)	0.022 (2)	0.0039 (16)	0.0078 (18)	0.0047 (16)
C20	0.053 (3)	0.039 (2)	0.036 (3)	0.010 (2)	0.024 (2)	−0.002 (2)
C21	0.042 (3)	0.045 (3)	0.045 (3)	−0.002 (2)	0.001 (2)	−0.007 (2)
C22	0.029 (2)	0.032 (2)	0.039 (3)	−0.0052 (18)	0.006 (2)	−0.013 (2)
C23	0.021 (2)	0.029 (2)	0.031 (3)	−0.0017 (16)	0.0086 (19)	−0.0007 (18)
C24	0.029 (2)	0.032 (2)	0.036 (3)	−0.0048 (18)	0.009 (2)	−0.0009 (19)
C25	0.024 (2)	0.036 (2)	0.046 (3)	0.0008 (18)	0.017 (2)	0.009 (2)
C26	0.033 (2)	0.036 (2)	0.032 (3)	0.0048 (19)	0.016 (2)	0.005 (2)
C27	0.029 (2)	0.034 (2)	0.032 (3)	−0.0038 (18)	0.011 (2)	−0.0010 (19)
C28	0.022 (2)	0.028 (2)	0.027 (2)	0.0033 (16)	0.0098 (19)	0.0010 (17)
C29	0.025 (2)	0.026 (2)	0.026 (2)	−0.0021 (17)	0.0105 (19)	−0.0027 (17)
C30	0.022 (2)	0.034 (2)	0.028 (3)	−0.0014 (17)	0.0065 (18)	−0.0018 (18)
C31	0.035 (3)	0.070 (4)	0.033 (3)	0.009 (2)	0.010 (2)	−0.004 (3)
C32	0.024 (2)	0.031 (2)	0.022 (2)	0.0010 (17)	0.0075 (18)	−0.0015 (17)
C33	0.024 (2)	0.031 (2)	0.021 (2)	−0.0018 (17)	0.0076 (19)	−0.0002 (17)
C34	0.024 (2)	0.029 (2)	0.022 (2)	−0.0007 (16)	0.0098 (18)	0.0036 (17)
C35	0.030 (2)	0.028 (2)	0.028 (3)	−0.0010 (18)	0.006 (2)	−0.0034 (18)
C36	0.025 (2)	0.036 (2)	0.031 (3)	−0.0079 (18)	0.011 (2)	−0.0009 (19)
C37	0.023 (2)	0.029 (2)	0.032 (3)	−0.0024 (17)	0.0091 (19)	0.0015 (18)
C38	0.029 (2)	0.038 (2)	0.021 (2)	−0.0023 (18)	0.0105 (19)	0.0017 (18)
C39	0.024 (2)	0.042 (2)	0.023 (2)	−0.0081 (18)	0.0045 (19)	−0.0015 (19)
C40	0.032 (2)	0.045 (3)	0.030 (3)	0.000 (2)	0.014 (2)	0.003 (2)
C41	0.040 (3)	0.055 (3)	0.049 (3)	0.005 (2)	−0.003 (3)	0.020 (3)
C42	0.051 (3)	0.060 (3)	0.049 (4)	0.023 (3)	0.007 (3)	0.011 (3)
C43	0.029 (2)	0.033 (2)	0.042 (3)	0.0004 (19)	0.011 (2)	−0.005 (2)
C44	0.026 (2)	0.038 (3)	0.061 (4)	0.006 (2)	0.013 (2)	−0.010 (2)
C45	0.029 (3)	0.042 (3)	0.055 (4)	−0.003 (2)	0.022 (3)	−0.012 (2)
C46	0.033 (3)	0.036 (2)	0.040 (3)	−0.007 (2)	0.019 (2)	−0.009 (2)
C47	0.025 (2)	0.034 (2)	0.034 (3)	−0.0042 (17)	0.013 (2)	−0.0061 (19)
C48	0.024 (2)	0.024 (2)	0.042 (3)	0.0000 (17)	0.011 (2)	−0.0051 (19)
C49	0.024 (2)	0.031 (2)	0.026 (2)	−0.0007 (17)	0.0055 (19)	−0.0028 (18)
C50	0.032 (2)	0.033 (2)	0.032 (3)	0.0062 (18)	0.008 (2)	0.0034 (19)
C51	0.045 (3)	0.071 (4)	0.038 (3)	−0.014 (3)	0.020 (3)	0.004 (3)
C52	0.023 (2)	0.034 (2)	0.018 (2)	−0.0021 (17)	0.0062 (18)	−0.0038 (17)
C53	0.025 (2)	0.030 (2)	0.024 (2)	−0.0033 (17)	0.0068 (19)	−0.0005 (17)
C54	0.026 (2)	0.026 (2)	0.028 (2)	−0.0001 (16)	0.0134 (19)	−0.0020 (17)
C55	0.028 (2)	0.030 (2)	0.019 (2)	0.0020 (17)	0.0092 (18)	0.0022 (17)
C56	0.023 (2)	0.032 (2)	0.026 (2)	0.0034 (17)	0.0041 (18)	0.0037 (18)
C57	0.026 (2)	0.024 (2)	0.026 (2)	0.0021 (16)	0.0129 (19)	−0.0044 (17)
C58	0.033 (2)	0.035 (2)	0.028 (3)	0.0020 (19)	0.011 (2)	−0.0029 (19)
C59	0.025 (2)	0.039 (2)	0.023 (2)	0.0039 (18)	0.0070 (19)	0.0001 (18)
C60	0.032 (2)	0.048 (3)	0.027 (3)	0.004 (2)	0.017 (2)	0.003 (2)
C61	0.059 (3)	0.053 (3)	0.024 (3)	0.014 (3)	0.010 (2)	0.009 (2)
C62	0.026 (2)	0.039 (2)	0.027 (3)	0.0029 (19)	0.004 (2)	0.0094 (19)
C63	0.029 (2)	0.027 (2)	0.026 (2)	0.0003 (17)	0.0127 (19)	0.0006 (17)
C64	0.027 (2)	0.029 (2)	0.032 (3)	0.0014 (17)	0.008 (2)	0.0025 (18)
C65	0.029 (2)	0.029 (2)	0.036 (3)	0.0023 (18)	0.014 (2)	−0.0001 (19)
C66	0.030 (2)	0.034 (2)	0.024 (2)	−0.0018 (18)	0.0130 (19)	−0.0024 (18)
C67	0.023 (2)	0.026 (2)	0.030 (3)	−0.0003 (16)	0.0092 (19)	0.0004 (17)
C68	0.021 (2)	0.027 (2)	0.022 (2)	−0.0018 (16)	0.0065 (17)	−0.0009 (16)
C69	0.021 (2)	0.030 (2)	0.019 (2)	−0.0021 (16)	0.0050 (17)	0.0013 (16)
C70	0.023 (2)	0.031 (2)	0.024 (2)	−0.0025 (17)	0.0078 (18)	−0.0027 (18)
C71	0.057 (3)	0.037 (3)	0.030 (3)	0.005 (2)	0.012 (2)	−0.005 (2)
C72	0.017 (2)	0.029 (2)	0.023 (2)	−0.0009 (15)	0.0067 (17)	−0.0007 (17)
C73	0.027 (2)	0.031 (2)	0.018 (2)	0.0021 (17)	0.0082 (18)	−0.0001 (17)
C74	0.022 (2)	0.027 (2)	0.026 (2)	−0.0008 (16)	0.0077 (18)	−0.0038 (17)
C75	0.026 (2)	0.030 (2)	0.028 (2)	−0.0008 (17)	0.0088 (19)	0.0026 (18)
C76	0.029 (2)	0.027 (2)	0.029 (3)	0.0013 (17)	0.009 (2)	0.0047 (18)
C77	0.024 (2)	0.032 (2)	0.026 (2)	0.0012 (17)	0.0063 (18)	−0.0032 (18)
C78	0.024 (2)	0.033 (2)	0.025 (2)	0.0033 (17)	0.0063 (18)	0.0009 (17)
C79	0.029 (2)	0.028 (2)	0.026 (2)	0.0030 (17)	0.0110 (19)	0.0002 (17)
C80	0.058 (3)	0.039 (3)	0.033 (3)	0.010 (2)	0.023 (2)	−0.002 (2)

### Geometric parameters (Å, °)

**Table:**

O1—C6	1.391 (5)	C26—C27	1.375 (6)
O1—C11	1.422 (6)	C27—C28	1.412 (6)
O2—C12	1.244 (5)	C27—H27	0.9500
O3—C13	1.219 (5)	C28—C29	1.448 (6)
O4—C17	1.378 (5)	C29—C30	1.394 (6)
O4—C20	1.414 (5)	C29—C32	1.430 (6)
O5—C26	1.369 (5)	C30—H30	0.9500
O5—C31	1.422 (6)	C31—H31A	0.9800
O6—C32	1.242 (5)	C31—H31B	0.9800
O7—C33	1.232 (5)	C31—H31C	0.9800
O8—C37	1.381 (5)	C32—C33	1.545 (6)
O8—C40	1.440 (5)	C34—C35	1.387 (6)
O9—C46	1.374 (6)	C34—C39	1.389 (6)
O9—C51	1.424 (7)	C35—C36	1.369 (6)
O10—C52	1.239 (5)	C35—H35	0.9500
O11—C53	1.224 (5)	C36—C37	1.383 (6)
O12—C57	1.372 (5)	C36—H36	0.9500
O12—C60	1.425 (5)	C37—C38	1.377 (6)
O13—C66	1.371 (5)	C38—C39	1.397 (6)
O13—C71	1.423 (6)	C38—H38	0.9500
O14—C72	1.232 (5)	C39—H39	0.9500
O15—C73	1.228 (5)	C40—H40A	0.9800
O16—C77	1.370 (5)	C40—H40B	0.9800
O16—C80	1.410 (5)	C40—H40C	0.9800
N1—C10	1.344 (5)	C41—C42	1.506 (8)
N1—C3	1.407 (5)	C41—H41A	0.9800
N1—C2	1.465 (5)	C41—H41B	0.9800
N2—C13	1.337 (5)	C41—H41C	0.9800
N2—C14	1.413 (5)	C42—H42A	0.9900
N2—H2	0.88 (3)	C42—H42B	0.9900
N3—C30	1.339 (5)	C43—C44	1.390 (7)
N3—C23	1.406 (6)	C43—C48	1.399 (7)
N3—C22	1.468 (5)	C44—C45	1.371 (8)
N4—C33	1.334 (5)	C44—H44	0.9500
N4—C34	1.421 (5)	C45—C46	1.396 (7)
N4—H4C	0.85 (5)	C45—H45	0.9500
N5—C50	1.356 (6)	C46—C47	1.394 (6)
N5—C43	1.399 (6)	C47—C48	1.396 (6)
N5—C42	1.458 (6)	C47—H47	0.9500
N6—C53	1.354 (5)	C48—C49	1.451 (6)
N6—C54	1.431 (5)	C49—C50	1.395 (6)
N6—H6	0.96 (5)	C49—C52	1.427 (6)
N7—C70	1.349 (5)	C50—H50	0.9500
N7—C63	1.401 (5)	C51—H51A	0.9800
N7—C62	1.459 (5)	C51—H51B	0.9800
N8—C73	1.354 (5)	C51—H51C	0.9800
N8—C74	1.412 (5)	C52—C53	1.547 (6)
N8—H8	0.92 (3)	C54—C59	1.383 (6)
C1—C2	1.505 (7)	C54—C55	1.390 (6)
C1—H1A	0.9800	C55—C56	1.387 (6)
C1—H1B	0.9800	C55—H55	0.9500
C1—H1C	0.9800	C56—C57	1.388 (6)
C2—H2A	0.9900	C56—H56	0.9500
C2—H2B	0.9900	C57—C58	1.380 (6)
C3—C4	1.384 (6)	C58—C59	1.394 (6)
C3—C8	1.404 (6)	C58—H58	0.9500
C4—C5	1.378 (6)	C59—H59	0.9500
C4—H4	0.9500	C60—H60A	0.9800
C5—C6	1.398 (6)	C60—H60B	0.9800
C5—H5	0.9500	C60—H60C	0.9800
C6—C7	1.378 (6)	C61—C62	1.506 (7)
C7—C8	1.399 (6)	C61—H61A	0.9800
C7—H7	0.9500	C61—H61B	0.9800
C8—C9	1.450 (6)	C61—H61C	0.9800
C9—C10	1.398 (6)	C62—H62A	0.9900
C9—C12	1.425 (6)	C62—H62B	0.9900
C10—H10	0.9500	C63—C64	1.393 (6)
C11—H11A	0.9800	C63—C68	1.399 (6)
C11—H11B	0.9800	C64—C65	1.372 (6)
C11—H11C	0.9800	C64—H64	0.9500
C12—C13	1.550 (6)	C65—C66	1.410 (6)
C14—C15	1.387 (6)	C65—H65	0.9500
C14—C19	1.393 (6)	C66—C67	1.394 (6)
C15—C16	1.378 (6)	C67—C68	1.395 (6)
C15—H15	0.9500	C67—H67	0.9500
C16—C17	1.374 (6)	C68—C69	1.460 (6)
C16—H16	0.9500	C69—C70	1.402 (6)
C17—C18	1.392 (6)	C69—C72	1.435 (6)
C18—C19	1.391 (6)	C70—H70	0.9500
C18—H18	0.9500	C71—H71A	0.9800
C19—H19	0.9500	C71—H71B	0.9800
C20—H20A	0.9800	C71—H71C	0.9800
C20—H20B	0.9800	C72—C73	1.547 (6)
C20—H20C	0.9800	C74—C79	1.384 (6)
C21—C22	1.521 (6)	C74—C75	1.403 (6)
C21—H21A	0.9800	C75—C76	1.377 (6)
C21—H21B	0.9800	C75—H75	0.9500
C21—H21C	0.9800	C76—C77	1.386 (6)
C22—H22A	0.9900	C76—H76	0.9500
C22—H22B	0.9900	C77—C78	1.391 (6)
C23—C24	1.387 (6)	C78—C79	1.404 (6)
C23—C28	1.392 (6)	C78—H78	0.9500
C24—C25	1.385 (6)	C79—H79	0.9500
C24—H24	0.9500	C80—H80A	0.9800
C25—C26	1.406 (6)	C80—H80B	0.9800
C25—H25	0.9500	C80—H80C	0.9800
			
C6—O1—C11	117.4 (3)	C37—C36—H36	119.5
C17—O4—C20	116.9 (3)	C38—C37—O8	124.7 (4)
C26—O5—C31	116.5 (4)	C38—C37—C36	119.5 (4)
C37—O8—C40	116.9 (3)	O8—C37—C36	115.7 (4)
C46—O9—C51	117.7 (4)	C37—C38—C39	119.6 (4)
C57—O12—C60	117.3 (3)	C37—C38—H38	120.2
C66—O13—C71	117.6 (3)	C39—C38—H38	120.2
C77—O16—C80	117.5 (3)	C34—C39—C38	120.7 (4)
C10—N1—C3	108.7 (3)	C34—C39—H39	119.7
C10—N1—C2	128.6 (4)	C38—C39—H39	119.7
C3—N1—C2	122.7 (3)	O8—C40—H40A	109.5
C13—N2—C14	125.6 (4)	O8—C40—H40B	109.5
C13—N2—H2	116 (3)	H40A—C40—H40B	109.5
C14—N2—H2	118 (3)	O8—C40—H40C	109.5
C30—N3—C23	108.8 (4)	H40A—C40—H40C	109.5
C30—N3—C22	126.4 (4)	H40B—C40—H40C	109.5
C23—N3—C22	124.8 (4)	C42—C41—H41A	109.5
C33—N4—C34	127.0 (4)	C42—C41—H41B	109.5
C33—N4—H4C	122 (4)	H41A—C41—H41B	109.5
C34—N4—H4C	111 (4)	C42—C41—H41C	109.5
C50—N5—C43	108.9 (4)	H41A—C41—H41C	109.5
C50—N5—C42	127.9 (4)	H41B—C41—H41C	109.5
C43—N5—C42	123.0 (4)	N5—C42—C41	113.9 (4)
C53—N6—C54	126.4 (4)	N5—C42—H42A	108.8
C53—N6—H6	114 (3)	C41—C42—H42A	108.8
C54—N6—H6	118 (3)	N5—C42—H42B	108.8
C70—N7—C63	108.9 (4)	C41—C42—H42B	108.8
C70—N7—C62	127.6 (4)	H42A—C42—H42B	107.7
C63—N7—C62	123.5 (4)	C44—C43—C48	122.4 (5)
C73—N8—C74	124.6 (4)	C44—C43—N5	129.1 (5)
C73—N8—H8	116 (5)	C48—C43—N5	108.4 (4)
C74—N8—H8	119 (5)	C45—C44—C43	117.5 (5)
C2—C1—H1A	109.5	C45—C44—H44	121.2
C2—C1—H1B	109.5	C43—C44—H44	121.2
H1A—C1—H1B	109.5	C44—C45—C46	121.0 (5)
C2—C1—H1C	109.5	C44—C45—H45	119.5
H1A—C1—H1C	109.5	C46—C45—H45	119.5
H1B—C1—H1C	109.5	O9—C46—C47	123.8 (5)
N1—C2—C1	113.0 (4)	O9—C46—C45	114.2 (4)
N1—C2—H2A	109.0	C47—C46—C45	121.9 (5)
C1—C2—H2A	109.0	C46—C47—C48	117.2 (4)
N1—C2—H2B	109.0	C46—C47—H47	121.4
C1—C2—H2B	109.0	C48—C47—H47	121.4
H2A—C2—H2B	107.8	C47—C48—C43	119.9 (4)
C4—C3—C8	123.0 (4)	C47—C48—C49	133.7 (4)
C4—C3—N1	129.0 (4)	C43—C48—C49	106.3 (4)
C8—C3—N1	108.0 (3)	C50—C49—C52	127.5 (4)
C5—C4—C3	117.0 (4)	C50—C49—C48	106.6 (4)
C5—C4—H4	121.5	C52—C49—C48	125.4 (4)
C3—C4—H4	121.5	N5—C50—C49	109.8 (4)
C4—C5—C6	120.6 (4)	N5—C50—H50	125.1
C4—C5—H5	119.7	C49—C50—H50	125.1
C6—C5—H5	119.7	O9—C51—H51A	109.5
C7—C6—O1	123.8 (4)	O9—C51—H51B	109.5
C7—C6—C5	122.7 (4)	H51A—C51—H51B	109.5
O1—C6—C5	113.5 (4)	O9—C51—H51C	109.5
C6—C7—C8	117.3 (4)	H51A—C51—H51C	109.5
C6—C7—H7	121.4	H51B—C51—H51C	109.5
C8—C7—H7	121.4	O10—C52—C49	122.9 (4)
C7—C8—C3	119.3 (4)	O10—C52—C53	118.0 (4)
C7—C8—C9	134.0 (4)	C49—C52—C53	119.1 (4)
C3—C8—C9	106.7 (4)	O11—C53—N6	125.6 (4)
C10—C9—C12	126.6 (4)	O11—C53—C52	123.6 (4)
C10—C9—C8	105.9 (4)	N6—C53—C52	110.8 (4)
C12—C9—C8	127.5 (4)	C59—C54—C55	120.0 (4)
N1—C10—C9	110.7 (4)	C59—C54—N6	122.9 (4)
N1—C10—H10	124.6	C55—C54—N6	117.1 (4)
C9—C10—H10	124.6	C56—C55—C54	119.6 (4)
O1—C11—H11A	109.5	C56—C55—H55	120.2
O1—C11—H11B	109.5	C54—C55—H55	120.2
H11A—C11—H11B	109.5	C55—C56—C57	120.6 (4)
O1—C11—H11C	109.5	C55—C56—H56	119.7
H11A—C11—H11C	109.5	C57—C56—H56	119.7
H11B—C11—H11C	109.5	O12—C57—C58	125.8 (4)
O2—C12—C9	123.1 (4)	O12—C57—C56	114.7 (4)
O2—C12—C13	117.8 (4)	C58—C57—C56	119.5 (4)
C9—C12—C13	119.0 (4)	C57—C58—C59	120.2 (4)
O3—C13—N2	124.8 (4)	C57—C58—H58	119.9
O3—C13—C12	122.2 (4)	C59—C58—H58	119.9
N2—C13—C12	113.0 (4)	C54—C59—C58	120.0 (4)
C15—C14—C19	118.5 (4)	C54—C59—H59	120.0
C15—C14—N2	119.2 (4)	C58—C59—H59	120.0
C19—C14—N2	122.3 (4)	O12—C60—H60A	109.5
C16—C15—C14	120.8 (4)	O12—C60—H60B	109.5
C16—C15—H15	119.6	H60A—C60—H60B	109.5
C14—C15—H15	119.6	O12—C60—H60C	109.5
C17—C16—C15	120.4 (4)	H60A—C60—H60C	109.5
C17—C16—H16	119.8	H60B—C60—H60C	109.5
C15—C16—H16	119.8	C62—C61—H61A	109.5
C16—C17—O4	116.4 (4)	C62—C61—H61B	109.5
C16—C17—C18	120.1 (4)	H61A—C61—H61B	109.5
O4—C17—C18	123.4 (4)	C62—C61—H61C	109.5
C19—C18—C17	119.1 (4)	H61A—C61—H61C	109.5
C19—C18—H18	120.5	H61B—C61—H61C	109.5
C17—C18—H18	120.5	N7—C62—C61	114.5 (4)
C18—C19—C14	120.9 (4)	N7—C62—H62A	108.6
C18—C19—H19	119.5	C61—C62—H62A	108.6
C14—C19—H19	119.5	N7—C62—H62B	108.6
O4—C20—H20A	109.5	C61—C62—H62B	108.6
O4—C20—H20B	109.5	H62A—C62—H62B	107.6
H20A—C20—H20B	109.5	C64—C63—C68	123.0 (4)
O4—C20—H20C	109.5	C64—C63—N7	128.0 (4)
H20A—C20—H20C	109.5	C68—C63—N7	109.0 (4)
H20B—C20—H20C	109.5	C65—C64—C63	117.3 (4)
C22—C21—H21A	109.5	C65—C64—H64	121.4
C22—C21—H21B	109.5	C63—C64—H64	121.4
H21A—C21—H21B	109.5	C64—C65—C66	120.9 (4)
C22—C21—H21C	109.5	C64—C65—H65	119.5
H21A—C21—H21C	109.5	C66—C65—H65	119.5
H21B—C21—H21C	109.5	O13—C66—C67	124.4 (4)
N3—C22—C21	111.3 (4)	O13—C66—C65	114.3 (4)
N3—C22—H22A	109.4	C67—C66—C65	121.3 (4)
C21—C22—H22A	109.4	C66—C67—C68	118.0 (4)
N3—C22—H22B	109.4	C66—C67—H67	121.0
C21—C22—H22B	109.4	C68—C67—H67	121.0
H22A—C22—H22B	108.0	C67—C68—C63	119.3 (4)
C24—C23—C28	122.5 (4)	C67—C68—C69	135.0 (4)
C24—C23—N3	129.5 (4)	C63—C68—C69	105.7 (4)
C28—C23—N3	108.0 (4)	C70—C69—C72	127.6 (4)
C25—C24—C23	117.1 (4)	C70—C69—C68	106.6 (4)
C25—C24—H24	121.5	C72—C69—C68	125.8 (4)
C23—C24—H24	121.5	N7—C70—C69	109.8 (4)
C24—C25—C26	121.3 (4)	N7—C70—H70	125.1
C24—C25—H25	119.3	C69—C70—H70	125.1
C26—C25—H25	119.3	O13—C71—H71A	109.5
O5—C26—C27	124.4 (4)	O13—C71—H71B	109.5
O5—C26—C25	114.3 (4)	H71A—C71—H71B	109.5
C27—C26—C25	121.3 (5)	O13—C71—H71C	109.5
C26—C27—C28	117.9 (4)	H71A—C71—H71C	109.5
C26—C27—H27	121.0	H71B—C71—H71C	109.5
C28—C27—H27	121.0	O14—C72—C69	124.5 (4)
C23—C28—C27	119.8 (4)	O14—C72—C73	118.3 (4)
C23—C28—C29	106.7 (4)	C69—C72—C73	117.2 (4)
C27—C28—C29	133.5 (4)	O15—C73—N8	124.2 (4)
C30—C29—C32	127.5 (4)	O15—C73—C72	123.0 (4)
C30—C29—C28	105.9 (4)	N8—C73—C72	112.7 (4)
C32—C29—C28	126.3 (4)	C79—C74—C75	119.0 (4)
N3—C30—C29	110.5 (4)	C79—C74—N8	122.5 (4)
N3—C30—H30	124.7	C75—C74—N8	118.5 (4)
C29—C30—H30	124.7	C76—C75—C74	120.4 (4)
O5—C31—H31A	109.5	C76—C75—H75	119.8
O5—C31—H31B	109.5	C74—C75—H75	119.8
H31A—C31—H31B	109.5	C75—C76—C77	120.6 (4)
O5—C31—H31C	109.5	C75—C76—H76	119.7
H31A—C31—H31C	109.5	C77—C76—H76	119.7
H31B—C31—H31C	109.5	O16—C77—C76	115.4 (4)
O6—C32—C29	121.8 (4)	O16—C77—C78	124.7 (4)
O6—C32—C33	117.8 (4)	C76—C77—C78	119.9 (4)
C29—C32—C33	120.4 (4)	C77—C78—C79	119.4 (4)
O7—C33—N4	126.3 (4)	C77—C78—H78	120.3
O7—C33—C32	122.4 (4)	C79—C78—H78	120.3
N4—C33—C32	111.3 (4)	C74—C79—C78	120.7 (4)
C35—C34—C39	118.7 (4)	C74—C79—H79	119.6
C35—C34—N4	118.0 (4)	C78—C79—H79	119.6
C39—C34—N4	123.3 (4)	O16—C80—H80A	109.5
C36—C35—C34	120.5 (4)	O16—C80—H80B	109.5
C36—C35—H35	119.8	H80A—C80—H80B	109.5
C34—C35—H35	119.8	O16—C80—H80C	109.5
C35—C36—C37	121.0 (4)	H80A—C80—H80C	109.5
C35—C36—H36	119.5	H80B—C80—H80C	109.5
			
C10—N1—C2—C1	−5.9 (6)	C50—N5—C42—C41	9.4 (8)
C3—N1—C2—C1	173.9 (4)	C43—N5—C42—C41	−164.2 (5)
C10—N1—C3—C4	−179.5 (4)	C50—N5—C43—C44	−177.9 (5)
C2—N1—C3—C4	0.7 (6)	C42—N5—C43—C44	−3.2 (8)
C10—N1—C3—C8	1.0 (4)	C50—N5—C43—C48	−1.7 (5)
C2—N1—C3—C8	−178.8 (3)	C42—N5—C43—C48	173.0 (5)
C8—C3—C4—C5	1.1 (6)	C48—C43—C44—C45	0.3 (7)
N1—C3—C4—C5	−178.3 (4)	N5—C43—C44—C45	176.1 (5)
C3—C4—C5—C6	0.8 (6)	C43—C44—C45—C46	−0.3 (7)
C11—O1—C6—C7	10.1 (6)	C51—O9—C46—C47	−2.9 (7)
C11—O1—C6—C5	−170.5 (4)	C51—O9—C46—C45	177.9 (4)
C4—C5—C6—C7	−2.4 (7)	C44—C45—C46—O9	179.8 (4)
C4—C5—C6—O1	178.2 (4)	C44—C45—C46—C47	0.5 (7)
O1—C6—C7—C8	−178.7 (4)	O9—C46—C47—C48	−179.9 (4)
C5—C6—C7—C8	2.0 (6)	C45—C46—C47—C48	−0.8 (7)
C6—C7—C8—C3	−0.1 (6)	C46—C47—C48—C43	0.8 (6)
C6—C7—C8—C9	179.1 (4)	C46—C47—C48—C49	−177.1 (5)
C4—C3—C8—C7	−1.5 (6)	C44—C43—C48—C47	−0.6 (7)
N1—C3—C8—C7	178.1 (3)	N5—C43—C48—C47	−177.1 (4)
C4—C3—C8—C9	179.1 (4)	C44—C43—C48—C49	177.8 (4)
N1—C3—C8—C9	−1.4 (4)	N5—C43—C48—C49	1.3 (5)
C7—C8—C9—C10	−178.1 (4)	C47—C48—C49—C50	177.6 (5)
C3—C8—C9—C10	1.2 (4)	C43—C48—C49—C50	−0.4 (5)
C7—C8—C9—C12	−0.4 (7)	C47—C48—C49—C52	5.3 (8)
C3—C8—C9—C12	178.9 (4)	C43—C48—C49—C52	−172.7 (4)
C3—N1—C10—C9	−0.3 (5)	C43—N5—C50—C49	1.4 (5)
C2—N1—C10—C9	179.6 (4)	C42—N5—C50—C49	−173.0 (5)
C12—C9—C10—N1	−178.4 (4)	C52—C49—C50—N5	171.5 (4)
C8—C9—C10—N1	−0.6 (4)	C48—C49—C50—N5	−0.6 (5)
C10—C9—C12—O2	176.1 (4)	C50—C49—C52—O10	−172.5 (4)
C8—C9—C12—O2	−1.2 (7)	C48—C49—C52—O10	−1.8 (7)
C10—C9—C12—C13	−6.2 (6)	C50—C49—C52—C53	6.0 (6)
C8—C9—C12—C13	176.5 (4)	C48—C49—C52—C53	176.7 (4)
C14—N2—C13—O3	−1.6 (7)	C54—N6—C53—O11	8.6 (7)
C14—N2—C13—C12	177.9 (4)	C54—N6—C53—C52	−170.6 (4)
O2—C12—C13—O3	172.9 (4)	O10—C52—C53—O11	−169.8 (4)
C9—C12—C13—O3	−4.9 (6)	C49—C52—C53—O11	11.6 (6)
O2—C12—C13—N2	−6.6 (5)	O10—C52—C53—N6	9.3 (5)
C9—C12—C13—N2	175.6 (4)	C49—C52—C53—N6	−169.2 (4)
C13—N2—C14—C15	−147.2 (5)	C53—N6—C54—C59	−29.9 (7)
C13—N2—C14—C19	33.9 (6)	C53—N6—C54—C55	151.0 (4)
C19—C14—C15—C16	1.2 (7)	C59—C54—C55—C56	3.0 (6)
N2—C14—C15—C16	−177.7 (4)	N6—C54—C55—C56	−177.9 (4)
C14—C15—C16—C17	1.1 (8)	C54—C55—C56—C57	−0.8 (6)
C15—C16—C17—O4	179.3 (5)	C60—O12—C57—C58	9.7 (6)
C15—C16—C17—C18	−2.6 (8)	C60—O12—C57—C56	−169.8 (4)
C20—O4—C17—C16	−163.1 (4)	C55—C56—C57—O12	177.8 (4)
C20—O4—C17—C18	18.9 (7)	C55—C56—C57—C58	−1.7 (6)
C16—C17—C18—C19	1.8 (7)	O12—C57—C58—C59	−177.5 (4)
O4—C17—C18—C19	179.7 (4)	C56—C57—C58—C59	1.9 (6)
C17—C18—C19—C14	0.6 (6)	C55—C54—C59—C58	−2.8 (6)
C15—C14—C19—C18	−2.0 (6)	N6—C54—C59—C58	178.1 (4)
N2—C14—C19—C18	176.9 (4)	C57—C58—C59—C54	0.3 (7)
C30—N3—C22—C21	−100.6 (5)	C70—N7—C62—C61	8.8 (6)
C23—N3—C22—C21	81.1 (5)	C63—N7—C62—C61	−171.6 (4)
C30—N3—C23—C24	176.6 (4)	C70—N7—C63—C64	178.4 (4)
C22—N3—C23—C24	−4.9 (7)	C62—N7—C63—C64	−1.3 (7)
C30—N3—C23—C28	−2.3 (5)	C70—N7—C63—C68	−2.0 (5)
C22—N3—C23—C28	176.2 (4)	C62—N7—C63—C68	178.3 (4)
C28—C23—C24—C25	−2.6 (7)	C68—C63—C64—C65	−1.0 (6)
N3—C23—C24—C25	178.6 (4)	N7—C63—C64—C65	178.6 (4)
C23—C24—C25—C26	−0.5 (7)	C63—C64—C65—C66	−2.1 (6)
C31—O5—C26—C27	−0.5 (7)	C71—O13—C66—C67	−12.7 (6)
C31—O5—C26—C25	179.9 (4)	C71—O13—C66—C65	167.9 (4)
C24—C25—C26—O5	−177.6 (4)	C64—C65—C66—O13	−176.7 (4)
C24—C25—C26—C27	2.7 (7)	C64—C65—C66—C67	3.9 (7)
O5—C26—C27—C28	178.6 (4)	O13—C66—C67—C68	178.2 (4)
C25—C26—C27—C28	−1.8 (7)	C65—C66—C67—C68	−2.4 (6)
C24—C23—C28—C27	3.6 (6)	C66—C67—C68—C63	−0.6 (6)
N3—C23—C28—C27	−177.4 (4)	C66—C67—C68—C69	179.5 (4)
C24—C23—C28—C29	−176.5 (4)	C64—C63—C68—C67	2.3 (6)
N3—C23—C28—C29	2.5 (5)	N7—C63—C68—C67	−177.3 (3)
C26—C27—C28—C23	−1.3 (6)	C64—C63—C68—C69	−177.7 (4)
C26—C27—C28—C29	178.8 (4)	N7—C63—C68—C69	2.6 (4)
C23—C28—C29—C30	−1.8 (5)	C67—C68—C69—C70	177.6 (4)
C27—C28—C29—C30	178.1 (5)	C63—C68—C69—C70	−2.3 (4)
C23—C28—C29—C32	172.9 (4)	C67—C68—C69—C72	0.0 (7)
C27—C28—C29—C32	−7.2 (8)	C63—C68—C69—C72	−179.9 (4)
C23—N3—C30—C29	1.1 (5)	C63—N7—C70—C69	0.4 (5)
C22—N3—C30—C29	−177.4 (4)	C62—N7—C70—C69	−179.9 (4)
C32—C29—C30—N3	−174.2 (4)	C72—C69—C70—N7	178.7 (4)
C28—C29—C30—N3	0.5 (5)	C68—C69—C70—N7	1.2 (4)
C30—C29—C32—O6	179.6 (4)	C70—C69—C72—O14	−173.5 (4)
C28—C29—C32—O6	6.0 (7)	C68—C69—C72—O14	3.5 (6)
C30—C29—C32—C33	0.1 (7)	C70—C69—C72—C73	7.5 (6)
C28—C29—C32—C33	−173.6 (4)	C68—C69—C72—C73	−175.5 (4)
C34—N4—C33—O7	−5.9 (7)	C74—N8—C73—O15	1.0 (7)
C34—N4—C33—C32	172.7 (4)	C74—N8—C73—C72	−176.3 (4)
O6—C32—C33—O7	174.4 (4)	O14—C72—C73—O15	−170.2 (4)
C29—C32—C33—O7	−6.1 (6)	C69—C72—C73—O15	8.9 (6)
O6—C32—C33—N4	−4.3 (5)	O14—C72—C73—N8	7.1 (5)
C29—C32—C33—N4	175.3 (4)	C69—C72—C73—N8	−173.8 (3)
C33—N4—C34—C35	−154.9 (4)	C73—N8—C74—C79	−34.3 (6)
C33—N4—C34—C39	24.4 (7)	C73—N8—C74—C75	147.7 (4)
C39—C34—C35—C36	−2.2 (6)	C79—C74—C75—C76	1.3 (6)
N4—C34—C35—C36	177.2 (4)	N8—C74—C75—C76	179.4 (4)
C34—C35—C36—C37	0.3 (7)	C74—C75—C76—C77	−0.1 (6)
C40—O8—C37—C38	−8.4 (6)	C80—O16—C77—C76	176.4 (4)
C40—O8—C37—C36	171.4 (4)	C80—O16—C77—C78	−3.0 (6)
C35—C36—C37—C38	1.8 (7)	C75—C76—C77—O16	179.6 (4)
C35—C36—C37—O8	−178.0 (4)	C75—C76—C77—C78	−1.0 (6)
O8—C37—C38—C39	178.0 (4)	O16—C77—C78—C79	−179.8 (4)
C36—C37—C38—C39	−1.8 (7)	C76—C77—C78—C79	0.8 (6)
C35—C34—C39—C38	2.1 (6)	C75—C74—C79—C78	−1.5 (6)
N4—C34—C39—C38	−177.2 (4)	N8—C74—C79—C78	−179.4 (4)
C37—C38—C39—C34	−0.1 (7)	C77—C78—C79—C74	0.5 (6)

### Hydrogen-bond geometry (Å, °)

**Table:**

*D*—H···*A*	*D*—H	H···*A*	*D*···*A*	*D*—H···*A*
N2—H2···O6^i^	0.88 (3)	2.17 (3)	2.967 (5)	150 (4)
N4—H4C···O2^ii^	0.85 (5)	2.44 (5)	3.245 (5)	159 (5)
N6—H6···O14^iii^	0.96 (5)	2.30 (5)	3.196 (5)	154 (4)
N8—H8···O10^iv^	0.92 (3)	2.08 (4)	2.936 (5)	155 (7)

Symmetry codes: (i) −*x*+1, *y*−1/2, −*z*+1; (ii) −*x*+1, *y*+1/2, −*z*+1; (iii) *x*, *y*, *z*+1; (iv) *x*, *y*, *z*−1.
